# Deficits in Visual System Functional Connectivity after Blast‐Related Mild TBI are Associated with Injury Severity and Executive Dysfunction

**DOI:** 10.1002/brb3.454

**Published:** 2016-03-23

**Authors:** Casey S. Gilmore, Jazmin Camchong, Nicholas D. Davenport, Nathaniel W. Nelson, Randy H. Kardon, Kelvin O. Lim, Scott R. Sponheim

**Affiliations:** ^1^ Defense and Veterans Brain Injury CenterMinneapolisMinnesota; ^2^ Minneapolis Veterans Affairs Health Care SystemMinneapolisMinnesota; ^3^ Department of PsychiatryUniversity of MinnesotaMinneapolisMinnesota; ^4^ Univ. of St. ThomasGraduate School of Professional PsychologyMinneapolisMinnesota; ^5^ Department of Ophthalmology & Visual ScienceUniversity of IowaIowa CityIowa; ^6^ Iowa City Veterans Affairs Health Care SystemIowa CityIowa

**Keywords:** blast TBI, executive function, functional connectivity, rest fMRI, TBI severity, traumatic brain injury, Visual

## Abstract

**Introduction:**

Approximately, 275,000 American service members deployed to Iraq or Afghanistan have sustained a mild traumatic brain injury (mTBI), with 75% of these incidents involving an explosive blast. Visual processing problems and cognitive dysfunction are common complaints following blast‐related mTBI.

**Methods:**

In 127 veterans, we examined resting fMRI functional connectivity (FC) of four key nodes within the visual system: lateral geniculate nucleus (LGN), primary visual cortex (V1), lateral occipital gyrus (LO), and fusiform gyrus (FG). Regression analyses were performed (i) to obtain correlations between time‐series from each seed and all voxels in the brain, and (ii) to identify brain regions in which FC variability was related to blast mTBI severity. Blast‐related mTBI severity was quantified as the sum of the severity scores assigned to each of the three most significant blast‐related injuries self‐reported by subjects. Correlations between FC and performance on executive functioning tasks were performed across participants with available behavioral data (*n* = 94).

**Results:**

Greater blast mTBI severity scores were associated with lower FC between: (A) LGN seed and (i) medial frontal gyrus, (ii) lingual gyrus, and (iii) right ventral anterior nucleus of thalamus; (B) V1 seed and precuneus; (C) LO seed and middle and superior frontal gyri; (D) FG seed and (i) superior and medial frontal gyrus, and (ii) left middle frontal gyrus. Finally, lower FC between visual network regions and frontal cortical regions predicted worse performance on the WAIS digit‐symbol coding task.

**Conclusion:**

These are the first published results that directly illustrate the relationship between blast‐related mTBI severity, visual pathway neural networks, and executive dysfunction – results that highlight the detrimental relationship between blast‐related brain injury and the integration of visual sensory input and executive processes.

## Introduction

Traumatic brain injury (TBI) is defined as an alteration in brain function, or evidence of other brain pathology, caused by an external force (Menon et al. 2010). In the U.S. military, TBI is a widespread problem. The Department of Defense reported that 333,169 service members sustained a TBI between 2000–2015, with 82% of those being classified as mild TBI (mTBI) (http://dvbic.dcoe.mil/dod-worldwide-numbers-tbi). One of the most common reported injuries to service members is mTBI from explosive blast, such as those from improvised explosive devices (Taber et al. 2006; Warden 2006). Of those veterans returning from the wars in Iraq and Afghanistan who experienced head injury, approximately 75% of these incidents involved an explosive blast (Hoge et al. 2008).

The mechanisms of blast‐related TBI are complex, and multiple components of the blast can cause injury. The notion that TBI might arise from primary blast exposure has been debated for many years (Denny‐Brown and Adams 1945; Säljö et al. 2011), and the precise mechanisms that might underlie blast‐related mTBI continue to be important points of theoretical discussion in the context of the recent wars in Iraq and Afghanistan (cf. Courtney and Courtney 2011). Research suggests that the primary effects of blast involve a series of pressure waves with compressive, tensile, and cavitation components that impose stress on the brain tissue (Taber et al. 2006; Moore and Jaffee 2010; Panzer et al. 2012). Additionally, injuries may occur from the secondary and tertiary effects of blast. Secondary blast injury results from being hit by debris put in motion by the blast wind, while tertiary blast injury results from a person being blown into solid objects or thrown against the ground (Taber et al. 2006; Elder and Cristian 2009). While the theoretical mechanisms of blast mTBI are complex and still not fully understood, it is hypothesized that the primary, secondary, and tertiary effects of blast exposure may be mediating factors to diffuse axonal injury (Hemphill et al. 2011; MacDonald et al. 2011), widespread white matter disruptions (Davenport et al. 2012; Taber et al. 2015), and damage to cerebral vasculature (Gama Sosa et al. 2014).

While blast‐related TBI has the potential to affect multiple sensory systems (e.g., the auditory system, Lew et al. 2007a), this study is driven by research implicating the visual system as susceptible to damage from the mechanisms of blast‐related mTBI (Elder et al. 2010; Dougherty et al. 2011; Magone et al. 2014). Blast‐related TBI may be accompanied by involvement of the visual system through optic nerve injury, diffuse or focal cerebral injury, or ocular motor disruption due to cranial nerve damage (Taber et al. 2006; Cockerham et al. 2009; Mohan et al. 2013; Magone et al. 2014). Any of these injuries mediated by blast‐related TBI mechanisms (including primary, secondary, and tertiary effects of blast exposure) may compromise the functional connectivity of visual neural pathways.

Evidence of alterations in visual function in individuals with blast‐related mTBI has been previously reported (Brahm et al. 2009; Goodrich et al. 2013; Magone et al. 2013). In a recent study of combat‐injured service members with reported histories of TBI, the majority of those with blast‐related mTBI reported visual complaints and presented with visual dysfunctions (Brahm et al. 2009). Visual dysfunctions in blast‐related TBI include retinal injuries, optic nerve damage, photosensitivity, oculomotor difficulties, and binocular vision deficits (Goodrich et al. 2007; Lew et al. 2007b; Cockerham et al. 2009; Mohan et al. 2013). Further, there is evidence that 68% of patients who reported a history of blast‐induced mTBI also reported visual complaints years after the injury (Magone et al. 2014).

Any injury that contributes to impairment in visual dysfunction (whether blast‐related mTBI or otherwise) has the potential to be substantially debilitating. It is estimated that much of the information stored in the human brain is directly or indirectly related to visual processes (De Moraes 2013). Critical information from the outside world enters the brain through the visual pathway, which integrates sensory information that is relayed to higher order executive processes (Zelinsky 2010). Blast‐related mTBI may disrupt proper information relay and subsequently affect perception, cognition, and behavior. Increased visual dysfunctions after blast‐related mTBI have been found to correlate with deficits in higher level processing such as reading speed and comprehension (Capó‐Aponte et al. 2012).

Disruption of information relay in blast‐related TBI can happen at different levels along the visual pathway from the retina to primary and secondary visual cortices (Cockerham et al. 2009). Major nodes in the visual pathway that may be affected by blast‐related TBI include (i) lateral geniculate nucleus (LGN), (ii) primary visual cortex (V1, striate cortex, or Brodmann's area 17), (iii) lateral occipital gyrus (LO), and fusiform gyrus (FG). LGN is located in the thalamus and acts as a relay of information primarily from retina to primary visual cortex (Schneider et al. 2004), but also receives modulatory input from the cortex (Murphy et al. 1999; Sherman and Guillery 2002). Diffusion tensor imaging (DTI) has shown the structural connectivity of thalamocortical tracts to be disrupted in TBI (Squarcina et al. 2012). V1 plays a critical role in visual information processing because most visual information bound for the rest of visual cortex first passes through V1 (Felleman and Van Essen 1991; Tootell et al. 1998). V1's location at the posterior pole of the occipital cortex makes it particularly susceptible to damage from impact and blast‐related TBI mechanisms (e.g., Wardlaw and Goeller 2010; Panzer et al. 2012). LO receives input from V1, and is involved in object recognition and categorization (Malach et al. 1995; Grill‐Spector et al. 2001). FG is part of the ventral stream anterior to the LO which responds preferentially to recognizable objects, most notably faces and words (Haxby et al. 1994), and is involved in visuospatial navigation (Jahn et al. 2004, 2009).

This study focused on examining brain functional organization of visual networks in a sample of veterans who reported histories of blast‐related mTBI. To investigate specific abnormalities in the visual pathway without the possibly confounding effects of stimulus presentation or task performance, this study examined the brain functional connectivity (FC) during rest and its relationship with the severity of blast‐related mTBI. The brain's intrinsic (resting) functional organization, which can be measured by examining the temporal coherence of resting‐state fluctuations, builds representations and updates information that serve as foundation for future responses to external stimuli (Mennes et al. 2010, 2011; Gour et al. 2011; Koyama et al. 2011; Zhu et al. 2011).

Resting FC can be highly consistent across healthy subjects (Moussa et al. 2012). It has been reported, however, that blast‐related injury can affect resting FC. Robinson et al. (2015) found decreased connectivity of regions in the most commonly examined resting state network, the default‐mode network, in close‐range blast exposure and blast‐related mTBI. Han et al. (2014) found spatially localized reductions in the participation coefficient, a measure of between‐module connectivity, in service members who had suffered a concussive blast‐related TBI. Sponheim et al. (2011) found reduced interhemispheric coordination of brain activity during rest, exhibited by diminished EEG phase synchrony of lateral frontal sites with contralateral frontal brain regions in individuals with self‐reported histories of blast exposure. This EEG phase synchrony was associated with the structural integrity of white matter tracts of the frontal lobe (Sponheim et al. 2011). The relationship between severity of blast‐related injury and resting FC within visual resting‐state networks, however, has not been examined. While these findings begin to identify alterations in resting functional organization in blast mTBI, more research is needed to further identify abnormalities specific to visual processing pathways.

Given the above literature, the main purpose of this study was to determine whether resting FC of regions within the visual pathway is related to blast‐related mTBI severity. Furthermore, based on evidence that the strength of functional connections of resting state networks is directly correlated with and can predict quality of executive performance (Debbané et al. 2012; Horowitz‐Kraus et al. 2015; Koyama et al. 2011; Markett et al. 2014; Mennes et al. 2010; Mennes et al. 2011; Reineberg et al. 2015; Xu et al. 2015), we examined the relationship between strength of resting FC of regions related to blast mTBI and performance in tasks that measure executive control. This study is the first to: (i) examine resting FC of specific nodes within the visual pathway in a sample of veterans, (ii) investigate whether resting FC alterations within the visual pathway differ as a function of the severity of blast mTBI, and (iii) explore whether identified resting FC alterations are related to behavior assessed outside of the scanner.

In this study we (i) examined resting FC of regions of interest that comprise key nodes in the visual pathway including lateral geniculate nucleus of the thalamus (LGN), primary visual cortex (V1), lateral occipital cortex (LO), and fusiform gyrus (FG); (ii) conducted linear regression analyses to identify the brain regions that have significant correlations between strength of resting FC and blast mTBI severity scores, (iii) conducted stepwise multiple regressions to examine the specificity of the relationship between blast mTBI severity and visual system FC in the presence of variables related to blast exposure and psychiatric comorbidity, and (iv) conducted correlations to examine the relationship between strength of resting FC of regions related to blast mTBI and performance in tasks that measure executive control (Stroop Color‐Word Test, Trail Making Test B, and the WAIS Digit‐Symbol Coding Task). Based on previous findings of deficits in sensory integration in mTBI (e.g., Stevens et al. 2012), we hypothesized that severity of blast mTBI would be negatively correlated with resting FC of regions of interest in the visual pathway. In addition, based on the hypothesis that the temporal coherence of resting fluctuations influence responses to external stimuli (Mennes et al. 2010, 2011; Gour et al. 2011; Koyama et al. 2011; Zhu et al. 2011), we expected to find a positive correlation between strength of resting FC, particularly between visual regions and prefrontal cortex (Tlustos et al. 2011), and successful performance on tasks involving executive function.

## Materials and Methods

### Participants

Participants consisted of 127 veterans of Operations Enduring and Iraqi Freedom (OEF/OIF), previously described (Davenport et al. 2014), with self‐reported traumatic combat experiences or exposure to explosive blasts during their most recent deployment (1–5 years before participation). Exclusion criteria included native language other than English, current or predeployment unstable medical condition that would reasonably be expected to significantly affect brain function (e.g., anoxic episode >10 sec, stroke, seizures, multiple sclerosis, etc.), uncorrected visual problems or hearing loss, moderate or severe TBI not due to blast, any predeployment Diagnostic and Statistical Manual of Mental Disorders, 4th Edition, Text Revision (DSM‐IV‐TR) (American Psychiatric Association, 2000) Axis I psychotic or mood disorder, current or past substance dependence other than nicotine or alcohol, and contraindications to MRI (e.g., metallic implants, shrapnel, and claustrophobia).

Participants provided written informed consent before enrollment in the study, and were compensated for participation after each study procedure. The study protocol was reviewed and approved by the University of Minnesota and Minneapolis Veterans Affairs Medical Center Institutional Review Boards and the U.S. Army Medical Research and Materiel Command.

Participants completed a clinical interview that included the Structured Clinical Interview for DSM‐IV‐TR (SCID; (First et al. 2002) and the Minnesota Blast Exposure Screening Tool (MN‐BEST; (Nelson et al. 2011). DSM‐IV‐TR diagnoses were assigned based on consensus review of all available information (SCID, medical records, behavioral observations, etc.) conducted by advanced doctoral students and doctoral‐level psychologists. Both “Current” (i.e., full diagnostic criteria met at the time of participation) and “Lifetime” (i.e., full diagnostic criteria met at any point in the individual's lifetime, past or present) diagnoses were considered. To assess the level of postconcussive symptoms (PCS) at the time of participation, participants were also asked whether they had experienced memory problems, poor balance, irritability, tinnitus, sensitivity to light/noise, headaches, or insomnia in the prior month. Intelligence Quotient (IQ) was estimated using the Wechsler Test of Adult Reading (WTAR) (Holdnack 2001).

### TBI assessment

Symptoms of mTBI were assessed by interview using the MN‐BEST and included altered consciousness (e.g., confusion and disorientation), loss of consciousness (LOC) less than 30 min, post‐traumatic amnesia (PTA) up to 24 h, and neurological symptoms (e.g., headache, tinnitus, nausea, sensitivity to light or noise) immediately after the event. Blast‐related injuries were defined as those in which the individual felt a blast wave and attributed the resultant concussion to its effects. Secondary blast effects, such as being hit by debris, were allowable as part of the overall blast‐related injury. Tertiary blast effects, such as being thrown against the ground, were acceptable provided that the blast wave itself was experienced as the source of those effects.

#### Blast TBI severity scoring

Ratings of TBI likelihood and severity were assigned by doctoral‐level neuropsychologists based on information secured by trained study interviewers using the MN‐BEST. The three most significant potential blast‐related TBI events were considered, each of which received a severity score ranging from 0 (no concussion) to a potential maximum of 30 (severe TBI), based on the severity rating scheme described below. No score was higher than 4 (the maximum within the mTBI range) in the current sample. Expanding upon the concussion severity rating scheme initially proposed by Ruff and Richardson (1999), concussions contributing to neurologic symptoms in the absence of LOC or PTA are rated as “Type 0” and assigned an overall blast‐related TBI score of “1”. Type I concussions are assigned an overall blast‐related TBI score of “2” and include ‘altered state or transient LOC’, PTA of no more than 60 sec and one or more neurologic symptoms. Type II and Type III concussions receive blast‐related TBI scores of “3” and “4”, respectively. Type II concussions consist of definite LOC of unknown duration to no more than 5 min, PTA from 60 sec to 12 h and at least one neurologic symptom. Type III concussions consist of complete LOC for 5 to no more than 30 min, PTA greater than 12 h and one or more neurologic symptoms. Based upon this scheme, the total blast‐related TBI severity score (called the Blast TBI Severity Index) for mild uncomplicated blast TBI is quantified as the sum of the scores of the three blast‐related events and ranges from 0 (no brain injury) to 12 (three Type III concussions) (Nelson et al. 2011). Non‐blast TBI events were also assessed using the same methodology.

### Imaging data acquisition

All participants underwent a 6‐min resting‐state fMRI scan and were instructed to be as still as possible, keep their eyes closed, and stay awake. Images were acquired on a 3 Tesla Siemens Trio (Erlagen, Germany) scanner using a 12‐channel birdcage head coil. Sequence parameters: gradient‐echo echo‐planar imaging (EPI) 180 volumes, repetition time (TR) = 2 sec, echo time (TE) = 30 ms, flip angle = 90°, 34 contiguous AC‐PC (anterior and posterior commissures) aligned axial slices with an interleaved acquisition, voxel size = 3.4 × 3.4× 4.0 mm, matrix = 64 × 64 × 34. Participants were debriefed at the end of the scan to confirm that they had stayed awake. A high‐resolution MP‐RAGE structural image (TR/TE = 2530/3.65 ms, 240 coronal slices, 256 × 256 matrix, 256 mm FOV, 1.0 mm thickness) was collected for anatomical alignment and visualization. A field map acquisition was collected and used to correct the fMRI data for geometric distortion caused by magnetic field inhomogeneities (TR = 300 ms, TE = 1.91 ms/4.37 ms, flip angle = 55°, voxel size = 3.4 × 3.4× 4.0 mm).

### FMRI imaging analysis

All individual‐level analyses (preprocessing and generation of FC maps) were conducted using the same procedures reported in previous studies (Camchong et al. 2014). The following prestatistics processing was applied for each subject using FEAT (FMRIB's Software Library (FSL)): first three volumes were deleted to account for magnetization stabilization, motion correction (FLIRT), B0 field map unwarping, slice‐timing correction, non‐brain removal (BET), spatial smoothing (with a 6‐mm full‐width half‐maximum kernel), grand mean scaling, high‐pass temporal filtering (100 Hz) to remove correlations associated with slow trends in scanner noise, and registration of all images to high‐resolution T1 and then to MNI (Montreal Neurological Institute) 2 × 2 × 2 mm standard space. Independent component analysis (ICA) was conducted for each individual to decompose individual data and conduct denoising by removing only the unique variance associated with components that account for noise while preserving the integrity of the continuous time‐series. Noise components included those that represented head motion (i.e., “rim‐like” artifacts around the brain, spikes in time‐series), scanner artifacts (i.e., slice dropouts, high‐frequency noise, field inhomogeneities), and physiological noise (i.e., respiration, cardiac frequencies, white matter signal, ventricular/cerebrospinal fluid fluctuations, frontal air cavities, ocular structures). Noise components were selected for removal using spatial and temporal characteristics detailed in the MELODIC (FSL) manual (http://www.fmrib.ox.ac.uk/fslcourse/lectures/melodic.pdf) and based on (Kelly et al. 2010) for selection criteria of noise components. Residual (denoised) data were computed by subtracting the selected noise components from the preprocessed data. Total variance accounting for head movement during rest fMRI scan (identified by ICA decomposition) was not significantly correlated with blast‐TBI severity (Spearman's *ρ* = −0.03, *P* = 0.72).

#### Regions of interest (ROI) selection and seed generation

**Figure 1:**
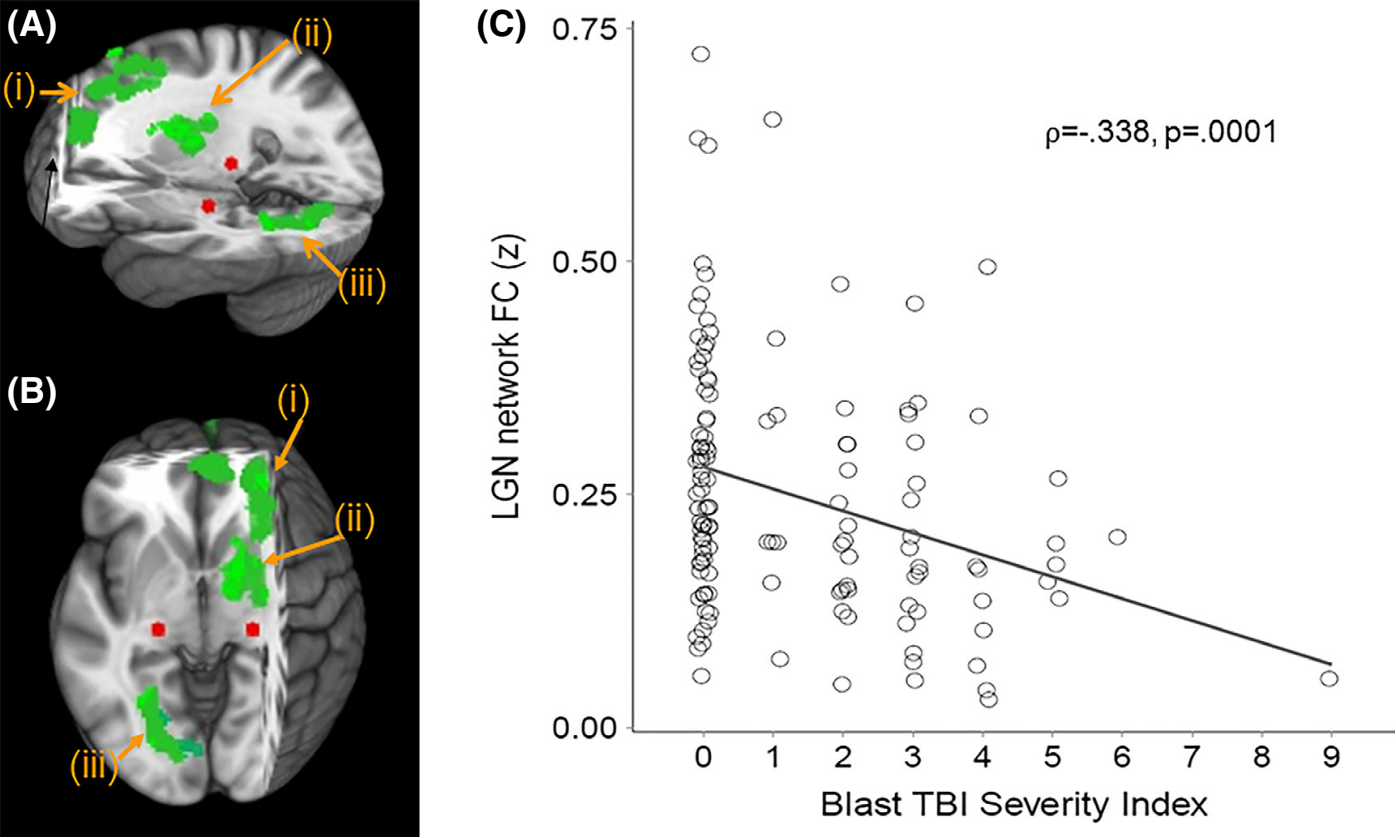
(A) Sagittal and (B) Axial views of three‐dimensional MNI brain with slices cut at *x* = −27, *y* = −3, *z* = −55, showing clusters with significant correlation with Blast mTBI Severity Scores and FC between lateral geniculate nucleus (LGN; red) and (i) dorsolateral and medial prefrontal cortex (BA 9 and 10), (ii) right ventral anterior nucleus of the thalamus, and (iii) lingual gyrus (BA 19). (C) Scatter plot showing significant correlation between Blast mTBI Severity Index scores and averaged LGN FC to green clusters (i, ii, and iii above). MNI, Montreal Neurological Institute; FC, functional connectivity; LGN, lateral geniculate nucleus; mTBI, Mild Traumatic brain injury; BA: Brodmann Area.

**Figure 2:**
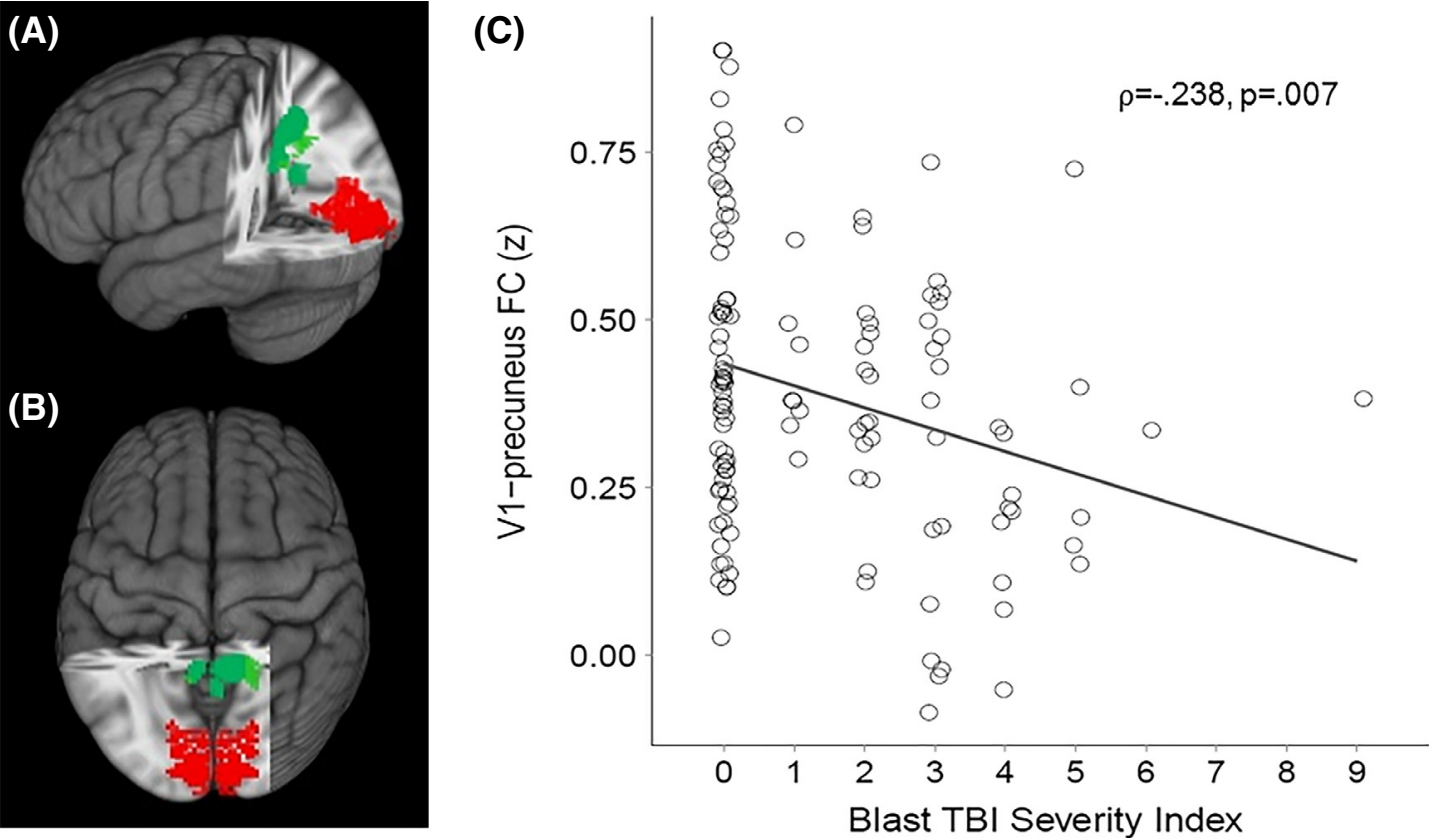
(A) Sagittal and (B) Axial views of three‐dimensional MNI brain with slices cut at *x* = −27, *y* = 46, *z* = −3, showing clusters with significant correlation between Blast mTBI Severity Scores and FC between primary visual cortex (BA17/V1; red) and precuneus (green). (C) Scatter plot showing significant correlation between Blast mTBI Severity Index scores and V1‐precuneus FC. MNI, Montreal Neurological Institute; FC, functional connectivity; mTBI, Mild Traumatic brain injury.

**Figure 3:**
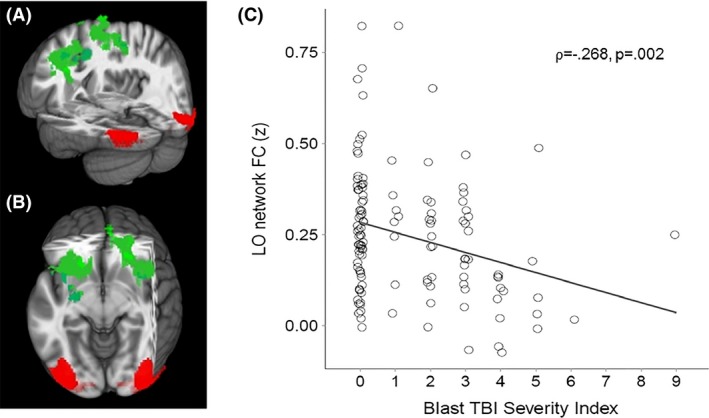
(A) Sagittal and (B) Axial views of three‐dimensional MNI brain with slices cut at *x* = −4, *y* = −34, *z* = −7, showing regions with significant correlation between Blast mTBI Severity Scores and FC between lateral occipital cortex (LO; red) and a frontal cluster comprised of bilateral middle and superior frontal gyri (BA 6; green). (C) Scatter plot showing significant correlation between Blast mTBI Severity Index scores and LO‐frontal FC. MNI, Montreal Neurological Institute; FC, functional connectivity; mTBI, Mild Traumatic brain injury; BA, Brodmann Area.

**Figure 4:**
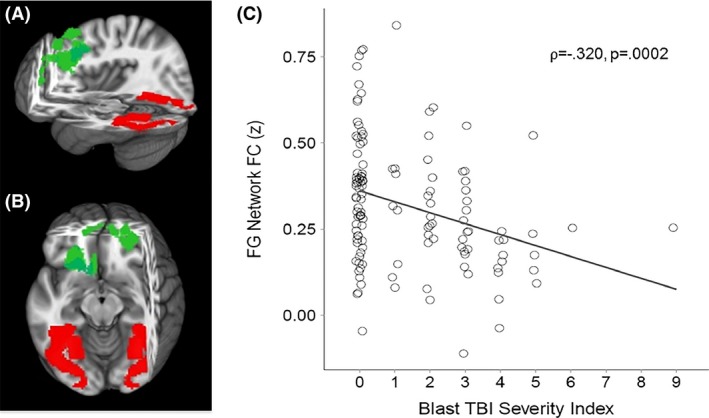
(A) Sagittal and (B) Axial views of three‐dimensional MNI brain with slices cut at *x* = −4, *y* = −34, *z* = −7, showing regions with significant correlation between Blast mTBI Severity Scores and FC between fusiform gyrus (FG; red) and a frontal cluster (green) comprised of superior and medial frontal gyri (BA 8 and 9, respectively). (C) Scatter plot showing significant correlation between Blast mTBI Severity Index scores and FG‐frontal FC. MNI, Montreal Neurological Institute; FC, functional connectivity; mTBI, Mild Traumatic brain injury; BA, Brodmann Area.

Because the purpose of this study was to examine the FC of key nodes in the visual pathway, we examined FC of lateral geniculate nucleus (LGN), primary visual cortex (V1), lateral occipital gyrus (LO), and fusiform gyrus (FG) (Figs. 1, 2, 3, 4, red areas). V1, LO, and FG seeds were defined by the Talairach Daemon atlas provided by AFNI (Analysis of Functional NeuroImages (Cox 2012). Because of its size, LGN seed was defined by using a spherical seed with 3.5 mm radius placed with center of mass in previously determined coordinates (Fujita et al. 2001). We extracted the time series from each seed for each participant by computing the mean intensity for all voxels within the seed region for each time point in the denoised residual data.

#### Resting state individual‐level analysis

A multiple regression analysis on the denoised data was performed between the extracted average time‐series from each seed and all voxels in the brain. This generated a map with a correlation coefficient (r) for each voxel, for each individual, for each seed. Correlation coefficients (r) were transformed to standardized *z* values. Resulting standardized *z‐*maps showed the degree of correlations with the corresponding seed averaged time‐series for each seed for each participant.

#### Resting state group‐level analysis

Linear regression analyses were conducted (AFNI – 3dRegAna program) to regress scores representing blast severity on the standardized z‐maps (for each seed separately). Resulting *F*‐statistic maps were used to identify brain regions in which individual FC variability was related to individual severity of blast. A threshold/cluster method derived from Monte Carlo simulations (AlphaSim, AFNI) was applied to control for false‐positive findings. Monte Carlo simulations (1000 iterations) accounted for the full‐width half‐maximum Gaussian filter (6 mm FWHM) and with a connectivity radius of 7.1 mm. On the basis of these simulations, the family‐wise *α* of 0.025 was preserved with an a priori voxel‐wise probability of 0.005 and three‐dimensional clusters with a minimum volume of 491 voxels. Clusters that survived correction for multiple comparison were identified (Figs. 1, 2, 3, 4, green areas).

### Behavioral tasks/Cognitive measures

A subset (*n* = 94) of participants performed the Stroop Color and Word test (Golden 1978), the Trail Making Test B (TMT; (Bowie and Harvey 2006)), and the Wechsler Adult Intelligence Scale Digit Symbol Coding subtest (WAIS‐III; (Wechsler 1997)) outside of the scanner. These tests were administered to participants to evaluate specific aspects of executive control, including cognitive efficiency, cognitive flexibility, and visual attention and memory (Golden 1978; Arbuthnott and Frank 2000; Lezak et al. 2004), that are potentially affected by improper visual sensory processing.

The Stroop Color‐Word Test includes three 45‐sec trials. Trial 1 entails rapid identification of simple repetitive words, while Trial 2 entails rapid identification of simple repetitive colors. The third and most challenging trial (the “Interference” trial) includes color words that are printed in incongruent ink (e.g., the word “red” printed in blue ink). Subjects must identify the color in which the word is printed, not the word itself. An Interference score was calculated in order to overcome the confound of performance on interference trials with subjects' facility for word reading. Interference score calculation is a function of scores from all three trial types (word, color, color‐word interference) and is detailed in Chafetz and Matthews (2004). Briefly, a predicted color‐word score is derived from the actual word and color trial scores, adjusted for the individual's ability to suppress word‐reading, and then subtracted from the actual score from the color‐word condition to give an Interference score (Chafetz and Matthews 2004). The Trail Making Test B requires a subject to “set shift”, or alternate attention, between numbers and letters (1, A, 2, B, etc.) to “connect‐the‐dots” of 25 consecutive targets on a sheet of paper. Scores represent the amount of time to complete the task. Longer times represent worse performance, as errors are identified and corrected during the trial, slowing down progress. The WAIS digit‐symbol coding task is a measure that involves the rapid identification of digit‐symbol pairs during a 2‐min trial. Scores represent the number of successfully completed pairings during the trial, which are then transformed to age‐adjusted scaled scores. Poor performance in these tasks has been previously found to be related to TBI severity (Langeluddecke and Lucas 2003; Demery et al. 2010).

#### FC correlates

The strength of resting FC (extracted *z*‐scores) within each network examined (LGN, V1, LO, FG seeds) was correlated with performance in the tasks mentioned above using Pearson's correlation coefficient (*r*). We applied a Holm–Bonferroni correction for multiple comparisons, controlling the family‐wise error rate at *P* = 0.05. Holm–Bonferroni (Holm 1979) is a sequential method in which the tests are first rank‐ordered from the one with the smallest *P*‐value to the one with the largest. The first test (the one with the smallest *P*‐value) is compared to *α*/the total number of tests, which here is 0.05/9 = 0.0056. If that test is significant, it is “removed”, then the second ranked test's *P*‐value is compared to *α*/the number of remaining tests (here, 0.05/8 = 0.0063), the third ranked test's *P*‐value is compared to 0.05/7, and so on, sequentially, for the remaining tests until a test is found to be not significant.

## Results

Demographic and clinical characteristics of the sample are described in Table 1.

**Table 1:** Demographics and clinical information of the sample

	Mean (SD)
Age	32.7 (8.1)
Men (% of sample)	92.1%
Education^a^	5.3 (0.7)
	Range: 4–7
WTAR estimated IQ^b^	105.0 (8.5)
Traumatic brain injury	
Blast TBI severity index	1.3 (1.7)
Time since most recent blast exposure (months)	47.6 (20.9)
Number of blast event exposures	25.3 (177.7)
	Median: 3.0
Total current PCS^c^	3.6 (2.5)
Non‐blast TBI severity index	1.9 (2.2)
Time since most recent non‐blast impact (months)	151.9 (99.7)
OEF/OIF deployments	
Number of deployments	1.4 (0.7)
Duration of deployments (months)	18.5 (7.2)
Time since most recent deployment (months)	36.4 (19.6)
Current psychiatric conditions^b^	
Post‐traumatic stress disorder	35.1%
Major depressive disorder	11.7%
Alcohol abuse/dependence	12.8%
Any axis I disorder	51.1%
Lifetime psychiatric conditions^b^	
Post‐traumatic stress disorder	45.7%
Major depressive disorder	39.4%
Alcohol abuse/dependence	40.4%
Any axis I disorder	67.0%
Current psychotropic medication usage^b^	
Antidepressants	22.8%
Mood Stabilizers	2.4%
Benzodiazepines	3.1%

SD, Standard Deviation; WTAR, Wechsler Test of Adult Reading; TBI, Traumatic Brain Injury; PCS, Post‐Concussive Symptoms.

a Education level was coded as follows: 1 = 7th Grade or less, 2 = Between 7th and 9th Grade, 3 = Between 10th and 12th, but not graduated, 4 = High School Graduate (includes GED), 5 = Partial College (includes business school, voc/tec), 6 = 4 year College/University Graduate, 7 = Graduate Degree (MA, PhD, MD, JD, etc.).

b These variables are reported for an *n* = 94 subset of subjects (i.e., data were unavailable for all subjects).

c A total of eight postconcussive symptoms were assessed: memory problems, poor balance, irritability, tinnitus, sensitivity to light, sensitivity to noise, headaches, and insomnia.

### Neuroimaging results

Given the non‐Gaussian distribution of the Blast TBI Severity Index scores, the nonparametric Spearman's rank‐order correlation coefficient (*⍴*) was used to identify regions in which blast mTBI severity scores were significantly correlated with resting functional connectivity (FC) of regions of interest (LGN, V1, LO, FG; Table 2). First, blast mTBI severity scores were found to be significantly correlated with FC between LGN and (i) dorsolateral prefrontal cortex (Brodmann areas ‐BA‐ 9 and 10), (ii) right ventral anterior nucleus of the thalamus, and (iii) lingual gyrus (BA 19)(*⍴* = −0.338, *P* = 0.0001; see Fig. 1). Second, blast mTBI severity scores were found to be significantly correlated with FC between primary visual cortex (V1) and precuneus (*⍴* = −0.238, *P* = 0.007; see Fig. 2). Third, blast mTBI severity scores were found to be significantly correlated with FC between lateral occipital cortex (LO) and superior/middle frontal cortex (BA 6)(*⍴* = −0.268, *P* = 0.002; see Fig. 3). Finally, blast mTBI severity scores were found to be significantly correlated with FC between fusiform gyrus (FG) and superior/medial frontal cortex (BA 8 and 9)(*⍴* = −0.320, *P* = 0.0002; see Fig. 4).

**Table 2:** MNI coordinates of identified clusters

			Center of Mass MNI Coordinates		
	Hemisphere	Brodmann Area	*x*	*y*	*z*
Lateral Geniculate Nucleus FC to:					
Dorsolateral and medial prefrontal cortex	Right	9 and 10	21	41	39
Thalamus, ventral anterior nucleus	Right		13	−7	16
Lingual gyrus	Left	19	−25	−71	−8
Primary Visual Cortex FC to:					
Precuneus	Right	7	10	−56	44
	Left	7	−7	−55	37
Lateral Occipital Cortex FC to:					
Middle frontal gyrus	Left	6	−36	4	49
	Right	6	39	1	45
Superior frontal gyrus	Right	6	19	14	56
Fusiform Gyrus FC to:					
Superior frontal gyrus	Right	8	22	24	58
Medial frontal gyrus	Left	9	−5	52	27

### Resting FC correlated with executive function

**Figure 5:**
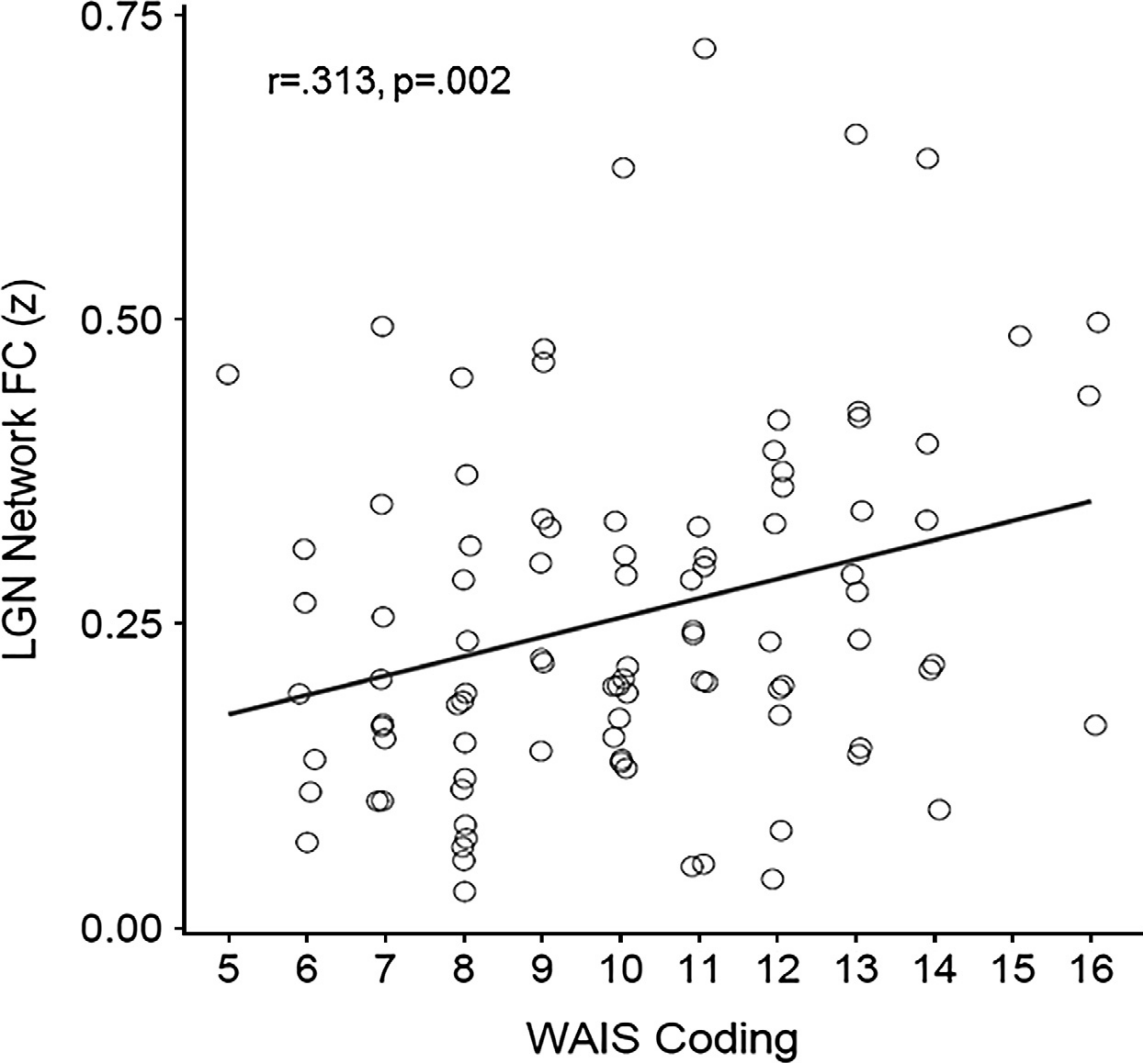
Scatter plot showing significant correlation between WAIS Digit‐Symbol Coding task performance scores and mean LGN FC (with regions listed in Table 2). WAIS Coding performance is measured as the number of successfully completed pairings during the trial, transformed to age‐adjusted Scaled Scores (more pairings = better performance). LO and FG FC showed similar relationships with WAIS Coding scores (these scatter plots are not shown).

We explored whether resting FC of the brain regions that showed significant correlations with TBI severity were related to task performance outside of the scanner. Because tasks administered assessed executive functioning, we limited our observations to identified resting FC networks that included frontal regions known to mediate executive control. Hence, average FC was calculated for LGN, LO, and FG networks. To correct for multiple comparisons, we applied a Holm–Bonferroni correction for nine total comparisons (3 resting FC networks that include frontal regions × 3 executive control tests), controlling the family‐wise error rate at *P* = 0.05. After correction, we found that the number of correct responses during the WAIS digit‐symbol coding task was significantly positively correlated (Pearson's *r*) with strength of FC between (i) LGN seed and DLPFC/medial prefrontal cortices, thalamus, and lingual gyrus (*r* = 0.313, *P* = 0.002), (ii) LO seed and middle/superior frontal gyri (*r* = 0.285, *P* = 0.005) and (iii) FG seed and superior/medial frontal gyri (*r* = 0.330, *P* = 0.001). Figure 5 illustrates correlation between WAIS Coding performance and LGN FC (correlations with FG and LO seeds showed same pattern). A similar correlation pattern was found between the FG network and Trails B task performance (*P*‐value < 0.05, however, significance did not survive correction for multiple comparisons); Stroop Interference score was not significantly correlated with FC in any network (see Table 3).

**Table 3:** Correlations (Pearson's r (*P*‐value)) between FC and task performance

	WAIS digit‐symbol coding	Stroop interference	Trails making B
LGN FC	**0.313 (0.002)**	0.087 (0.403)	−0.191 (0.065)
LO FC	**0.285 (0.005)**	0.044 (0.670)	−0.108 (0.302)
FG FC	**0.330 (0.001)**	0.030 (0.775)	−0.212 (0.041)

Total current PCS scores were negatively correlated with FC (LGN: *⍴* = −0.224, *P* = 0.013; V1: *⍴* = −0.178, *P* = 0.048; LO: *⍴* = −0.189, *P* = 0.035; FG: *⍴ *= −0.208, *P* = 0.021). However, these correlations were not significant after Holm–Bonferroni correction for multiple comparisons. Nor were there any significant correlations or observed trends between FC and (i) non‐blast mTBI severity scores, (ii) WTAR IQ, (iii) Current or Lifetime Axis I Disorders (based on point‐biserial correlations for diagnoses of PTSD, Alcohol Dependence, Major Depressive Disorder, or having any Axis I Disorder), (iv) OEF/OIF Deployment measures (number of deployments, duration of deployments, time since most recent deployment), or (v) time since most recent blast exposure.

### Blast mTBI severity and repeated blast exposures

As stated in the Methods section, during calculation of the MN‐BEST derived blast mTBI severity index score, severity ratings are summed across the three most significant blast mTBI events reported by the subject. As part of the TBI assessment procedure with the MN‐BEST, information is collected (via self‐report) on the number of exposures to blast events (regardless of whether an mTBI resulted; subjects are asked, “How many times did you feel the blast wave of an explosion?”) and on the number of those events (up to 3) that meet criteria for classification as an mTBI (as determined by a consensus group of experts). The three most significant blast‐related events are each given a severity rating, then these three severity scores are summed to get the MN‐BEST Blast TBI Severity Index score. This raises the potential concern that repeated mTBIs, or repeated exposure to blast (regardless of whether an mTBI resulted), may be a driving force behind the relationship between blast mTBI severity scores and visual system FC. In order to examine this possibility, stepwise multiple regressions were performed to evaluate whether blast mTBI severity remained the primary predictor of FC when other potentially relevant variables were also taken into account. We conducted stepwise multiple regressions including blast mTBI severity score, number of reported blast exposures, and number of blast mTBIs as predictors, and FC in each of the visual system networks as the criterion variable (separate multiple regressions were done for each of the four FC networks: LGN, V1, LO, and FG). Education level was also included as a predictor variable in the regression, as recent literature has shown relationships between education and resilience toward TBI (Kesler et al. 2003; Schneider et al. 2014; Holland and Schmidt 2015).

Table 4 shows the zero‐order Spearman's correlations between the variables included in the stepwise multiple regression analyses, as well as outcome measures, including the standardized *β*, F, and *t* test values, the multiple correlation coefficient *R*, and the amount of variance in the criterion variable accounted for by the variable included in the final model, *R* ^2^.

**Table 4:** Zero‐order correlations and stepwise multiple regression results of blast‐related variables, and education level, on functional connectivity in each visual system network

Zero‐order Spearmans *⍴* Correlations							
	Number of mTBIs	Number of blast exposures	Education	LGN	V1	LO	FG
Blast mTBI severity score	0.969***	0.492***	0.004	−0.338***	−0.238**	−0.268**	−0.320***
Number of mTBIs^a^		0.472***	−0.01	−0.314***	−0.221*	−0.233**	−0.297***
Number of blast exposures^a^			0.009	−0.183*	−0.175*	−0.141	−0.240**
Education				0.073	−0.035	0.059	0.103
	Stepwise multiple regression						
	*β*	*F*	*t*	*R*	*R* ^2^		
LGN model							
**Blast mTBI severity score**	−**0.336**	**15.96** ***		**0.336**	**0.113**		
Number of mTBIs			0.257				
Number of blast exposures			−0.658				
Education			1.468				
V1 model							
Blast mTBI severity score			−0.513				
**Number of mTBIs**	−**0.263**	**9.295** **		**0.263**	**0.069**		
Number of blast exposures			−0.176				
Education			−0.023				
LO model							
**Blast mTBI severity score**	−**0.277**	**10.37** **		**0.277**	**0.077**		
Number of mTBIs			0.554				
Number of blast exposures			−0.65				
Education			1.268				
FG model							
**Blast mTBI severity score**	−**0.307**	**13.017** ***		**0.307**	**0.094**		
Number of mTBIs			−0.278				
Number of blast exposures			−0.658				
Education			1.519				

*P < 0.05, **P < 0.01, ***P < 0.001.

Variables retained in each model are indicated in Bold print.

*β*, standardized beta weight; *R*, multiple correlation coefficient; *R* ^2^, proportion of variance explained.

a The “number of blast exposures” variable reflects the self‐reported estimation of number of blast events that involved feeling the pressure wave; these reports were taken at face value and not separately reviewed by consensus raters of blast mTBI. In contrast, “numbers of mTBI” were carefully reviewed by consensus raters.

Blast mTBI severity score and number of mTBIs showed significant zero‐order Spearman's correlations with FC in all four visual system networks; number of blast event exposures was significantly correlated with FC in LGN, V1, and FG networks. Education level was not significantly correlated with FC in any network.

Stepwise multiple regression showed that, for the LGN, LO, and FG networks, blast mTBI severity score alone significantly accounted for variance in FC. Neither the number of blast event exposures, number of mTBIs, nor Education accounted for any significant amount of variance in FC when entered into the model with blast mTBI severity. For the V1 seed network, number of reported blast mTBIs alone significantly accounted for variance in FC; the other variables did not account for a significant amount of variance in the presence of number of mTBIs. One caveat to this result, however, is the high correlation between blast TBI severity score and number of reported blast mTBIs (*⍴* = 0.969). To test for collinearity, the Tolerance statistic was calculated between these two measures. Tolerance was 0.145, which is just larger than the generally accepted value of 0.10 (values smaller than 0.10, on a scale of 0–1, indicating collinearity). This suggests that interpretation of the isolated effects of these variables, while valid, should be done with caution.

### Blast mTBI severity and psychiatric comorbidities

Finally, given the substantial number of psychiatric comorbidities and psychotropic medication usage present in the current sample (see Table 1), we tested the possibility that psychiatric comorbidity might be underlying the relationship between blast mTBI severity and FC. Stepwise multiple regressions were performed including the predictor variables blast mTBI severity score, presence of each of three lifetime axis I disorders (PTSD, Alcohol Dependence, or Major Depressive Disorder), presence of each of these axis I disorders currently, as well as a dichotomous measure of whether or not the subject was taking a psychotropic medication; FC in each of the visual system networks was the criterion variable (separate multiple regressions were done for each of the four FC networks: LGN, V1, LO, and FG). Only those subjects for whom psychiatric diagnosis and medication data was available (*n* = 94) were included in these analyses.

Table 5 shows the zero‐order Spearman's correlations between the variables included in the stepwise multiple regression analyses, as well as outcome measures (standardized *β*,* F*,* t*,* R*, and *R* ^2^). There were no significant zero‐order correlations between FC in any of the four visual system networks and any of the current or lifetime axis I disorders, nor medication usage. Stepwise multiple regression showed that, for each of the four visual system networks, blast mTBI severity score alone significantly accounted for variance in FC. Neither diagnosis of a lifetime axis I disorder, diagnosis of a current axis I disorder, nor psychotropic medication usage accounted for any significant amount of variance in FC when entered into the model with blast mTBI severity.

**Table 5:** Zero‐order correlations and stepwise multiple regression results of blast mTBI severity, psychiatric comorbidity, and psychotropic medication usage on functional connectivity in each visual system network

Zero‐order Spearmans *⍴* Correlations											
	Current PTSD	Lifetime PTSD	Current MDD	Lifetime MDD	Current ALC	Lifetime ALC	Medication Usage	LGN	V1	LO	FG
Blast mTBI severity score	0.310**	0.318**	0.310**	0.282**	0.148	0.279**	0.171	−0.361***	−0.259**	−0.313**	−0.354***
Current PTSD		0.801***	0.218*	0.320**	0.253*	0.439***	0.162	−0.137	−0.027	−0.056	−0.126
Lifetime PTSD			0.197	0.440***	0.225*	0.375***	0.201	−0.073	0.011	0.026	−0.032
Current MDD				0.452***	0.357***	0.172	−0.028	−0.088	−0.097	−0.138	−0.154
Lifetime MDD					0.214*	0.268**	0.060	−0.029	−0.070	−0.102	−0.095
Current ALC						0.464***	−0.121	−0.100	−0.019	0.096	0.041
Lifetime ALC							−0.005	−0.165	−0.042	−0.023	−0.086
Medication Usage								0.052	−0.026	−0.100	−0.011
	Stepwise Multiple Regression										
	*β*	*F*	*t*	*R*	*R* ^2^						
LGN model											
**Blast mTBI severity score**	**−0.362**	**13.915** ***		**0.362**	**0.131**						
Current PTSD			−0.896								
Lifetime PTSD			0.635								
Current MDD			0.925								
Lifetime MDD			1.298								
Current ALC			−0.67								
Lifetime ALC			−1.003								
Medication Usage			1.102								
V1 model											
**Blast mTBI severity score**	−**0.268**	**7.117** **		**0.268**	**0.072**						
Current PTSD			−0.11								
Lifetime PTSD			0.56								
Current MDD			−0.281								
Lifetime MDD			−0.073								
Current ALC			0.093								
Lifetime ALC			−0.156								
Medication Usage			0.125								
LO model											
**Blast mTBI severity score**	−**0.302**	**9.251** **		**0.302**	**0.091**						
Current PTSD			0.067								
Lifetime PTSD			1.466								
Current MDD			−0.582								
Lifetime MDD			0.278								
Current ALC			1.286								
Lifetime ALC			0.189								
Medication Usage			−0.145								
FG model											
**Blast mTBI severity score**	−**0.325**	**10.831** ***		**0.325**	**0.105**						
Current PTSD			−0.616								
Lifetime PTSD			0.945								
Current MDD			−0.917								
Lifetime MDD			0.299								
Current ALC			0.325								
Lifetime ALC			−0.619								
Medication Usage			0.256								

*P < 0.05, **P < 0.01, ***P < 0.001

Variables retained in each model are indicated in Bold print.

*β*, standardized beta weight; *R*, multiple correlation coefficient; *R*‐squared, proportion of variance explained PTSD, Post Traumatic Stress Disorder; MDD, Major Depressive Disorder; ALC, Alcohol Abuse/Dependence.

Only those subjects for whom psychiatric diagnosis and medication data was available (*n* = 94) were included in these analyses.

## Discussion

The overall objective of this study was to determine the relationship between blast‐related mTBI severity and the intrinsic functional connections of the brain's visual system. The study identified specific relationships such that the greater the blast‐related mTBI severity score, the lower the resting functional connectivity between key nodes in the visual pathway and frontal and parietal brain regions. Further, when accounting for the presence of other variables related to blast‐related exposure, injury, and psychiatric comorbidities, blast mTBI severity remained the primary determinant of strength of FC across visual system nodes in seven out of eight analyses. Finally, strength of functional connectivity within visual networks that included frontal areas was associated with performance on tests of executive functioning.

## Functional Connectivity of Visual Pathway Nodes

This study provided evidence that blast‐related mTBI severity is negatively correlated with FC of LGN with dorsolateral and medial prefrontal cortex (BA 9 and 10), right ventral anterior nucleus of the thalamus, and lingual gyrus. Thalamic dysfunction has been reported in mTBI (Ge et al. 2009; Little et al. 2010; Grossman et al. 2012; Squarcina et al. 2012), primarily in relation to structural integrity of the thalamus and thalamocortical projections. Damage to thalamic structural and functional connectivity may be an important factor in outcome after TBI (Squarcina et al. 2012), given its central role in information relay to and from the cortex. While thalamic disruption has been found in mTBI, this study is the first to report dysfunction specifically related to blast mTBI severity in LGN resting FC. Only one other study has examined resting state FC of the thalamus in subjects with mTBI. Tang et al. (2011) assessed a group of patients with closed head injury shortly after their injuries (average of 22 days). Resting state networks were identified between thalamus and frontal, temporal, and subcortical areas. Results showed that subjects with mTBI suffered disruption in thalamic resting state networks. Further, these thalamic functional connectivity abnormalities were associated with decreased performance on neurocognitive tests. Despite important methodological differences in the mTBI groups between Tang et al. (2011) and this study, results suggest a consistent pattern related to mTBI; FC of thalamic resting state networks are disrupted as a function of mTBI, and that disruption is related to performance on tests of executive functioning. Moreover, findings from this study provide important evidence regarding disease chronicity. Given that our sample had sustained their injuries years before participation in the study, this LGN FC disruption (as well as disruption in FC of the other networks we examined) may be a chronic condition. Longitudinal data would help detail the course of mTBI in military populations exposed to explosive blast.

Precuneus has been suggested to be a “core node” or “hub” of the default‐mode network (Fransson and Marrelec 2008), and it exhibits resting state FC with visual cortex (Margulies et al. 2009). Given the proposed role of precuneus in directing attention (Cavanna and Trimble 2006), and its strong interconnections with prefrontal cortex (Cavanna and Trimble 2006), this visual cortex/precuneus FC is likely part of a system involved in the regulation of visual attention (Lauritzen et al. 2009) – a system that may be affected by blast mTBI (Graner et al. 2013). The precuneus, per se, has shown both reduction in resting cerebral blood flow (Kim et al. 2010) and decreased regional volume (Zhou et al. 2013) as a function of severity of TBI, conditions that could contribute to current findings of reduced V1/precuneus FC with blast mTBI severity.

Interestingly, the V1/precuneus network was the only one in which the number of reported blast‐related mTBIs (up to 3, as assessed during the MN‐BEST) accounted for unique variance in FC in the presence of blast mTBI severity. As with blast mTBI severity, there was a negative correlation between number of reported blast mTBIs and FC in this network – more reported blast mTBIs were associated with reduced FC. This suggests that, for dysfunction in the V1/precuneus network, repeated blast‐related mTBIs may be an important factor to consider. Many of the psychological and physical effects of repetitive mTBI in the military population have been well studied (Vanderploeg et al. 2012; Bryan 2013; Bryan and Clemans 2013; Reid et al. 2014). The impact of multiple blast‐related mTBIs on the visual system, however, is unknown. The high correlation between blast TBI severity score and number of blast mTBIs in this study does not allow for strong inferences to be made about their isolated effects. Future research should be done on teasing apart the unique effects of repeated blast mTBI and blast mTBI severity on functional connectivity in the visual system.

Lateral occipital (LO) cortex and fusiform gyrus (FG) are both extrastriate regions that play key roles in visual object processing. LO receives input from V1, and is involved in object recognition and categorization (Malach et al. 1995; Grill‐Spector et al. 2001). FG is part of the ventral stream anterior to the LO which responds preferentially to recognizable objects, and is involved in visuospatial navigation (Jahn et al. 2004, 2009). In this study, both of these areas showed resting FC with partially overlapping frontal cortical areas. The strength of functional connectivity between these regions was negatively correlated with blast mTBI severity. These frontal areas – superior frontal, middle, and medial frontal gyri – are generally involved in executive control of behavior, for example attention, working memory, and planning (Nobre et al. 1997; Leung et al. 2002; Ranganath et al. 2003; Zhang et al. 2003; Kübler et al. 2006). Animal and human studies have shown that projections from visual cortical areas to medial prefrontal cortex support long‐term potentiation (Kim et al. 2003), and that visual perceptual learning is related to the strength of resting FC between visual cortical areas (including LO) and frontal and parietal areas involved in the control of visual attention (e.g., medial frontal) (Lewis et al. 2009). Structurally, both LO and FG are part of the inferior fronto‐occipital fasciculus (IFOF), a white matter associative bundle connecting occipital to frontal cortices (Caverzasi et al. 2014; Forkel et al. 2014; Sarubbo et al. 2015a,b). This fronto‐occipital connection formed by the IFOF has been found to support functions such as visuospatial attention and object recognition (Sarubbo et al. 2015b). While there is little information on any effects of TBI on networks including LO or FG specifically, studies of white matter integrity have shown lower white matter integrity (lower fractional anisotropy; FA) in IFOF in both non‐blast TBI as compared to controls (Bigler et al. 2010; Singh et al. 2010; Brandstack et al. 2013), as well as in blast mTBI compared to those with no blast injury (in a subset of the current subjects; (Davenport et al. 2012)). Further, studies have shown relationships between FA within the IFOF and performance on executive function tasks in mTBI (Kraus et al. 2007; Han et al. 2013). Our findings of an association between higher FC in LO and FG networks and better performance on a task that assesses executive function are in line with these findings.

Only one previous study has found a relationship specifically between mTBI and resting FC in visual system networks (Stevens et al. 2012). Using a group ICA analysis including both mTBI (non‐blast) and control subjects, they identified resting state functional networks, particularly one including primary visual cortex and one including secondary visual processing areas. The mTBI group showed deficits relative to controls in functional connectivity in the primary visual processing network, (which, like this study, included precuneus), while in the secondary visual processing network, enhancement of FC in mTBI was found. Further, similar to this study, Stevens et al. (2012) found correlations between FC within visual resting state networks and number of PCS in mTBI – more PCS were linked to less functional connectivity in the primary visual network – suggesting a relationship between mTBI severity and FC. It is important to note, however, that while this study suggests a unique relationship between primary visual system FC and blast‐related mTBI, Stevens et al. examined subjects with non‐blast mTBI (vs. controls). Methodological differences between the two studies do not allow an unequivocal statement that reduced FC in the primary visual system network is exclusively related to either blast or non‐blast mTBI. Thus, while our, and previous, findings suggest the existence of mTBI‐mediated FC alterations in visual pathway networks, the exact nature of this relationship merits further investigation.

## Executive Function and FC

As hypothesized, better performance on tasks assessing executive functioning was associated with greater FC in those visual system networks involving frontal cortical areas. While the association between FC and the Trails B task did not survive Holm–Bonferroni correction for multiple comparisons, the WAIS digit‐symbol coding task showed correlations across these networks. The Coding task has been shown to be highly sensitive to brain impairment (Russell 1972; Crowe et al. 1999). Cognitive abilities assessed by the Coding task include sustained attention (Lezak et al. 2004), working memory functions (Woo‐Sam et al. 1971), response speed (Salthouse 1992), perceptual organization (Kaufman 1990), and visual–motor coordination (Sprandel 1995) – all executive functions that have been shown to be affected by mTBI (McDonald et al. 2002; Demery et al. 2010). Current results indicate that the strength of resting state FC in identified visual‐frontal networks associated with blast‐related mTBI severity may be neural mechanisms that underlie neurocognitive abilities in mTBI.

While there was an association between Coding task performance and these resting FC measures, performance (the number of successfully completed digit‐symbol pairings during the trial) was not correlated with blast mTBI severity scores. This finding suggests that measures of neural function may be sensitive to the effects of blast‐related mTBI while behavioral measures of cognitive performance may fail to capture altered brain function due to the injury. It should be noted that because task performance was not measured in the scanner, a direct relationship between Coding performance and brain activity cannot be established based on current data. Given present and previous correlation results (Mennes et al. 2010; Tang et al. 2011; Zhu et al. 2011), however, there is evidence that synchrony of neural networks during rest is related to measurable behavior.

## Consequences of Blast mTBI on Visual Functions

Visual problems are one of the most common sequelae of blast‐related mTBI. Studies have reported that upwards of 66% of veterans with blast‐related TBI had subjective complaints about their vision (Goodrich et al. 2013), and that blast‐exposed veterans report significantly poorer visual quality compared to healthy controls (Lemke et al. 2013). Yet, most of these veterans had a normal eye exam using standardized tests of vision, visual field, and examination of the retina and optic nerve (Lemke et al. 2013). While standard eye tests did not indicate dysfunction, examination of neurons in the retina using non‐invasive retinal imaging by high‐resolution optical coherence tomography (OCT) revealed an almost 25% prevalence of significant loss of nerves in the inner layers of the retina of these veterans (Kardon et al. 2013). In studies on blast‐induced mild TBI in mice, evidence was found for a biphasic pattern of functional loss in the retina with progressive loss of retinal neurons over time (Mohan et al. 2013; Dutca et al. 2014). This study extends these findings from the retina into the thalamus and cortex, emphasizing the importance of examining the effects of blast mTBI on structure and function in the central nervous system in order to better characterize visual dysfunction. Such characterization allows us to differentiate between visual abnormalities that are direct effects of blast mTBI on the eye, retina, and optic nerve or secondary to trans‐synaptic retrograde degeneration from postgeniculate injury to the brain visual pathways.

## Caveats and Limitations

As with most studies conducted with OEF/OIF veteran samples, a limitation of this study relates to the fact that acute‐stage injury characteristics of blast‐related mTBI (e.g., LOC, post‐traumatic amnesia) were obtained exclusively on the basis of retrospective self‐report. Unfortunately, external records and other corroborating sources (e.g., eyewitness accounts) were not available for review, and this diminishes any understanding of the reliability and true severity of blast events encountered during combat. Indeed, researchers have shown that a significant proportion of OEF/OIF samples demonstrate inconsistencies in self‐reported combat‐related mTBI as the interval between combat participation and mTBI assessment increases (cf. Van Dyke et al. 2010; Polusny et al. 2011; Nelson et al. 2015). Although only 9% of OIF participants reported a history of combat‐related mTBI 1 month prior to returning to the U.S., rates of self‐reported combat‐related mTBI increased to 22% when the same participants were surveyed 1‐year postdeployment (Polusny et al. 2011). In a follow‐up study (Nelson et al. 2015), postdeployment symptoms of PTSD and diffuse health concerns were identified as the most significant predictors of inconsistent self‐reported mTBI over time. The latter results suggest that OEF/OIF veterans often experience difficulty representing previous blast and other combat events, and the true source(s) of any symptoms/impairments that may follow from these events (whether psychological, physiological, or both). Thus, present findings should be interpreted with these caveats about the reliability of self‐reported mTBI indices in mind.

The following caveats should also be kept in mind when considering these results. First, although subjects were debriefed at the end of the resting fMRI scan to find out whether they remained awake, they were not monitored with a periodic response or eye‐tracking. There is a risk that they were not truthful in their report. Second, although there is no definite evidence of functional connectivity differences during rest with eyes open versus eyes closed, we acknowledge that a more complete examination of the visual system during rest would have included an additional scan collected while subjects had their eyes open fixating on a cross (e.g., Patriat et al. 2013). Moreover, additional studies need to be conducted where identified abnormalities during rest are also probed while patients are performing a task that engages the visual network.

## Conclusion

The examination of resting FC is an important tool for examining the neurobiological sequelae of TBI. Taken together, current results point to the severity of blast mTBI impacting the degree of functional neural disconnection between nodes of the visual pathway and parietal and frontal cortical areas. Decreased FC as a function of blast mTBI severity suggests that blast‐related injuries may interfere with integration within functional brain networks – integration that is necessary for successful performance of both simple and complex tasks. Abnormal connectivity between the visual areas and frontal cortex may provide objective biomarkers of mTBI and underlie visual and cognitive impairment. The identification of underlying neural network abnormalities related to blast mTBI severity and cognitive dysfunction may guide targeted treatments developed to modulate FC within these networks.

## Conflict of Interest

None declared.
